# Correction: Twitter and Facebook posts about COVID-19 are less likely to spread misinformation compared to other health topics

**DOI:** 10.1371/journal.pone.0298907

**Published:** 2024-02-12

**Authors:** David A. Broniatowski, Daniel Kerchner, Fouzia Farooq, Xiaolei Huang, Amelia M. Jamison, Mark Dredze, Sandra Crouse Quinn, John W. Ayers

There are errors in the Funding section. The correct Funding statement is: This work was supported in part by grant number R01GM114771 to D.A. Broniatowski and S.C. Quinn, by grant number SES-2029420 to D. A. Broniatowski, and by the John S. and James L. Knight Foundation to the GW Institute for Data, Democracy, and Politics. The funders had no role in study design, data collection and analysis, decision to publish, or preparation of the manuscript.
