# Supplementary material for: Twitter and Facebook posts about COVID-19 are less likely to spread misinformation compared to other health topics
Source: PLoS One. 2022 Jan 12;17(1):e0261768. doi: 10.1371/journal.pone.0261768 (PMC8754324; doi:10.1371/journal.pone.0261768)
Supplement: S1 File. Appendix A — (PDF) [file pone.0261768.s001.pdf]

Unshortening URLs

For each data set, we identified all posts containing a URL, and extracted these URLs. Specifically, we used the "tldextract" Python module [1] to identify the top-level domain for each URL. Next, we identified URLs that had been shortened (e.g. "bit.ly/x11234b") by examining whether their top-level domains were present on a list of commonly-used URL shorteners that we compiled from several online sources:

- https://github.com/sambokai/ShortURL-Services-List
- https://gist.github.com/ninetyfivenorth/9322bfc20523ba2eb7521d57cf25f265
- https://github.com/738/awesome-url-shortener
- https://gist.github.com/BenderV/0fb893b034791337fb4e596759b761e2

We unshortened these links using the Python "requests" module [2] and then identified the TLD for each unshortened link. Next, we enumerated the frequency of each TLD in each dataset.

References

1. Kurkowski J. john-kurkowski/tldextract. 2020. Available: https://github.com/john-kurkowski/tldextract
2. Requests: HTTP for Humans™ — Requests 2.24.0 documentation. Available: https://requests.readthedocs.io/en/master/
