# Supplementary material for: Twitter and Facebook posts about COVID-19 are less likely to spread misinformation compared to other health topics
Source: PLoS One. 2022 Jan 12;17(1):e0261768. doi: 10.1371/journal.pone.0261768 (PMC8754324; doi:10.1371/journal.pone.0261768)
Supplement: S2 File — Measuring source credibility. (PDF) [file pone.0261768.s002.pdf]

Measuring source credibility

We obtained credibility ratings for each TLD using data compiled on July 5, 2020 by two independent sources:

1. Ratings from the NewsGuard database, licensed from NewsGuard. NewsGuard provides "detailed ratings of more than 4,500 news websites that account for 95% of online engagement with news." (https://www.NewsGuard.com/) For each website in its database, NewsGuard provides a composite rating from 0-100, constituting a sum across nine separate journalistic standards.
2. Ratings from the MediaBiasFactCheck website (https://mediabiasfactcheck.com/). MediaBiasFactCheck rates several TLDs according to their factual accuracy into six categories: "Very Low", "Low", "Mixed", "Mostly Factual", "High", and "Very High".

We found that 950 domains across all datasets were assigned ratings by both NewsGuard and MediaBiasFactCheck. Treating MediaBiasFactCheck factual accuracy ratings as a six-point Likert scale with increments of 20 (0="Very Low" to 100 = "Very High"), we found that NewsGuard and MediaBiasFactCheck scores were strongly correlated, $r(948)=0.81, p<0.001$. Following true score theory [1], this high correlation indicates that NewsGuard and MediaBiasFactCheck scores measure the same underlying construct – source credibility.

Our procedure for rating TLDs was as follows:

1. We coded as "government" all TLDs ending in .gov, .gc.ca, .mil, .nhs.uk, starting with gov., mygov., government., containing .govt. or .gov., or matching who.int, paho.org, un.org, canada.ca, ontario.ca, toronto.ca, or alberta.ca (n=6,913 domains).
2. We coded as "academic" all unscored TLDs ending in .edu, containing .edu., .ac., thelancet.com, sciencedirect.com., medrxiv.org, pnas.org, apa.org, nature.com, sciencemag.org, nejm.org, bmj.com, mayoclinic.org, aaas.org, healthdata.org, researchgate.net, or rand.org (n=5,116 domains).

3. NewsGuard coded several TLDs, including social media platforms such as reddit.com, and news aggregators such as eurekalert.com and medium.com, as “Platforms”. To this list, we added blogspot.com, facebook.com, instagram.com, linkedin.com, t.me, tiktok.com, twitter.com, t.co, and whatsapp.com. Any domains on this list were coded as “Platforms” in our analysis (n=37 domains).
4. For each TLD that was rated by both NewsGuard and MediaBiasFactCheck, we averaged these ratings (n=950 domains).
5. Among the remaining TLDs, several (n=3,563 domains) were given a numerical rating by either MediaBiasFactCheck or NewsGuard, but not both. In this case, we retained this single numerical rating.
6. Among the remaining unscored TLDs, several were categorized by either MediaBiasFactCheck or NewsGuard as “Satire” (n=30 domains), or by MediaBiasFactCheck as “Questionable” (indicating propaganda or fake news; n=5 domains). We retained these categories if no other numerical ratings were available.

We assigned each of these categories a credibility score: academic, government, and scaled news credibility scores exceeding 2/3 (66.67/100; n=3,149) were labeled as “More Credible”; scaled news credibility scores between 1/3 (33.33/100) and 2/3 (n=789) were labeled as “Less Credible”, and scaled news credibility scores less than 1/3 (n=575), and “Questionable” MediaBiasFactCheck scores (but no NewsGuard scores) were labeled as “Not Credible”. Categories that were not assigned to these three high-level groups, including platforms, satire sites, and all remaining domains, were labeled as “unrated”.

References

1. Lord FM. A strong true-score theory, with applications. Psychometrika. 1965;30: 239–270.
