# Supplementary material for: Twitter and Facebook posts about COVID-19 are less likely to spread misinformation compared to other health topics
Source: PLoS One. 2022 Jan 12;17(1):e0261768. doi: 10.1371/journal.pone.0261768 (PMC8754324; doi:10.1371/journal.pone.0261768)
Supplement: S3 File — Categorizing tweets with multiple URLs. (PDF) [file pone.0261768.s003.pdf]

Categorizing tweets with multiple URLs

Several tweets contained multiple URLs

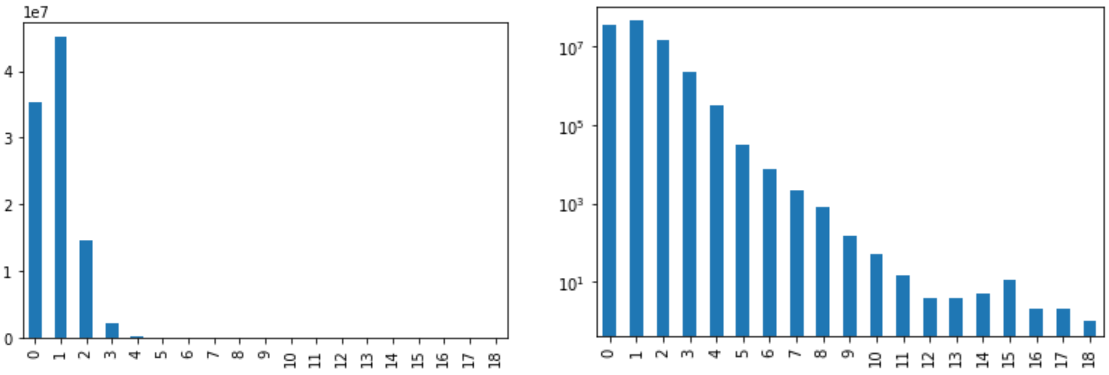

Distribution of URLs per tweet for original tweets in the COVID-19 Twitter dataset. Data are shown using linear (left) and logarithmic (right) scales.

These tweets were categorized in the following order of priority:

1. Not credible
2. Less Credible
3. Government Website
4. Academic
5. Other More Credible
