# Supplementary figures and images for: Twitter and Facebook posts about COVID-19 are less likely to spread misinformation compared to other health topics
Source: PLoS One. 2022 Jan 12;17(1):e0261768. doi: 10.1371/journal.pone.0261768 (PMC8754324; doi:10.1371/journal.pone.0261768)

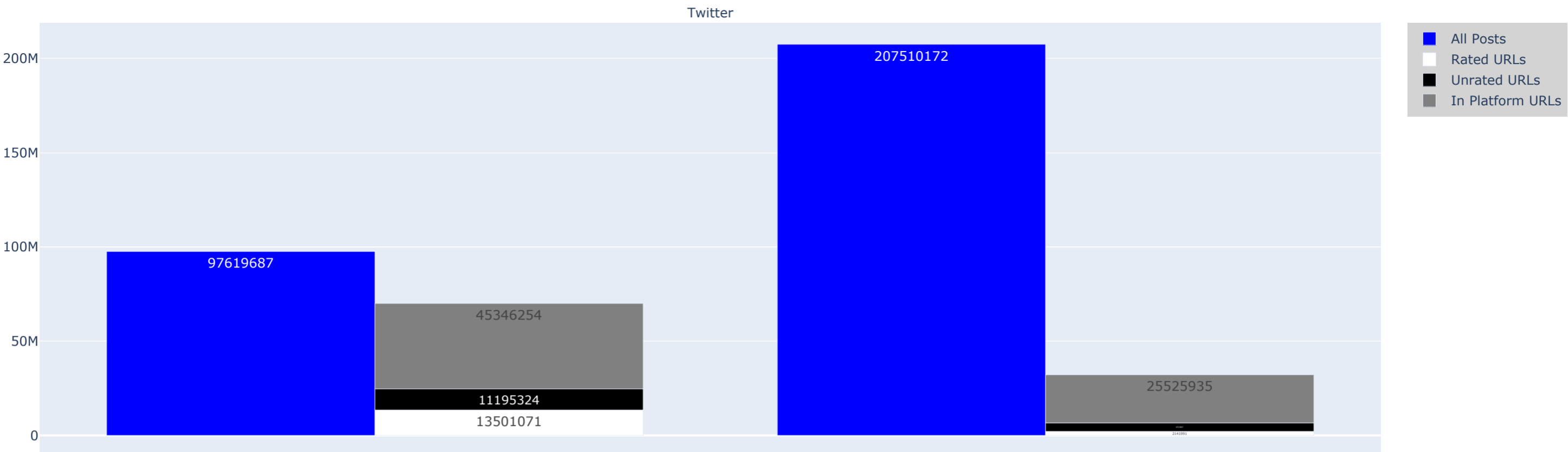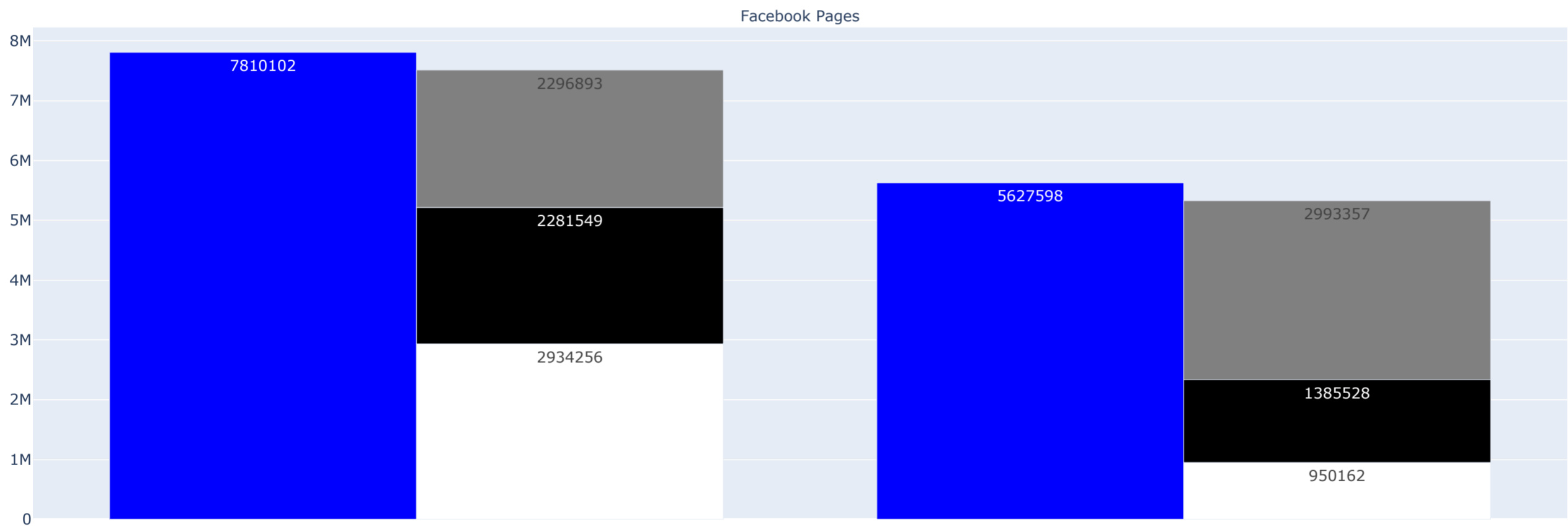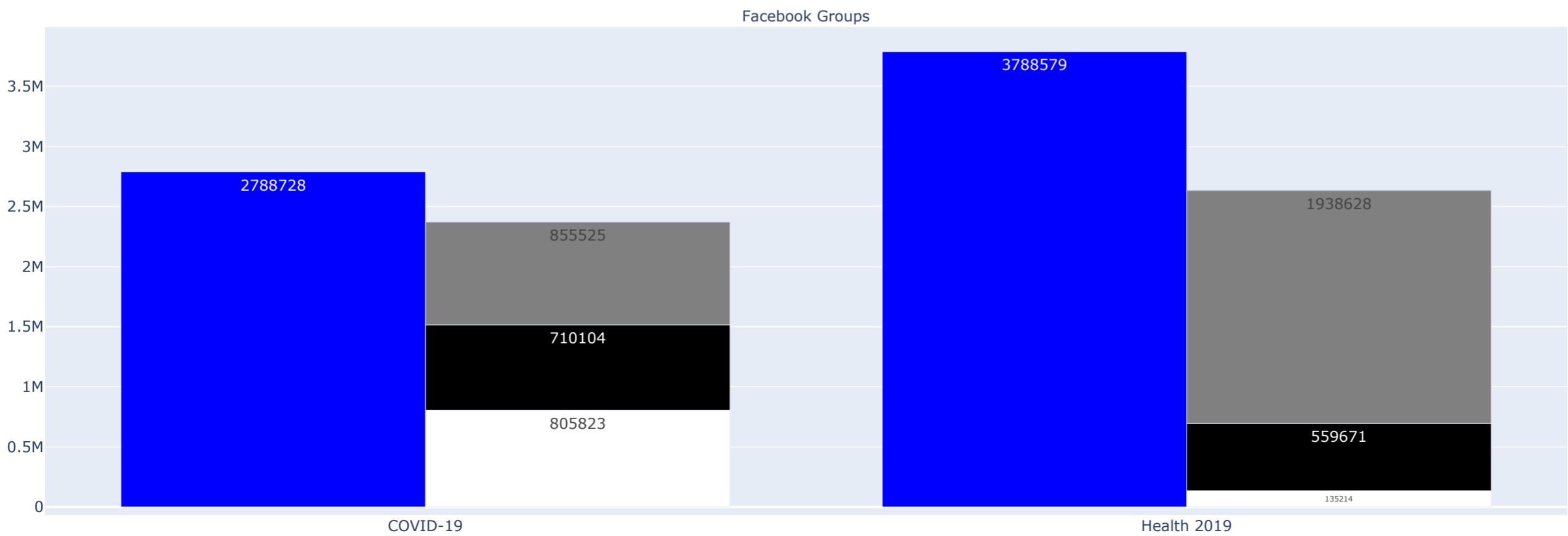

Supplement: S1 Fig — URLs are segmented by whether they were rated, unrated, or “in platform” (e.g., pointing from Facebook to Facebook or from Twitter to Twitter). (PDF) [file pone.0261768.s004.pdf]

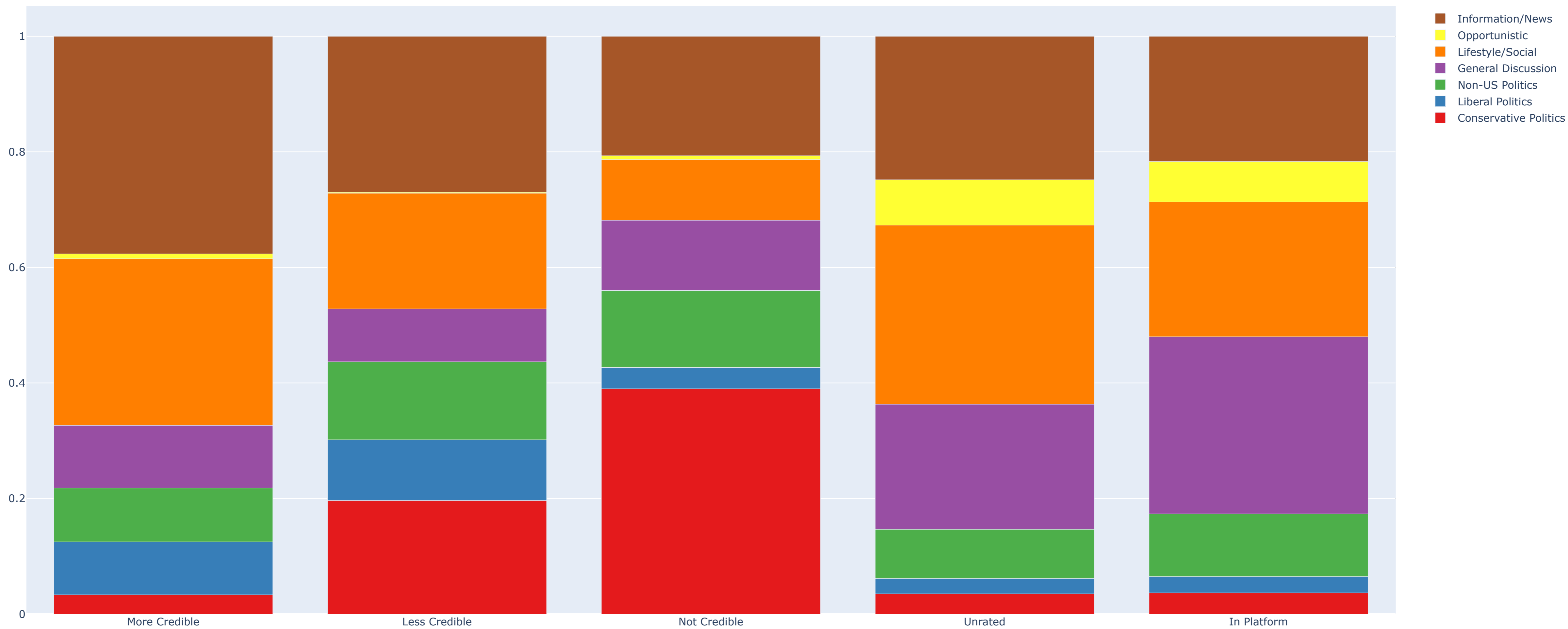

Supplement: S2 Fig — (PDF) [file pone.0261768.s005.pdf]

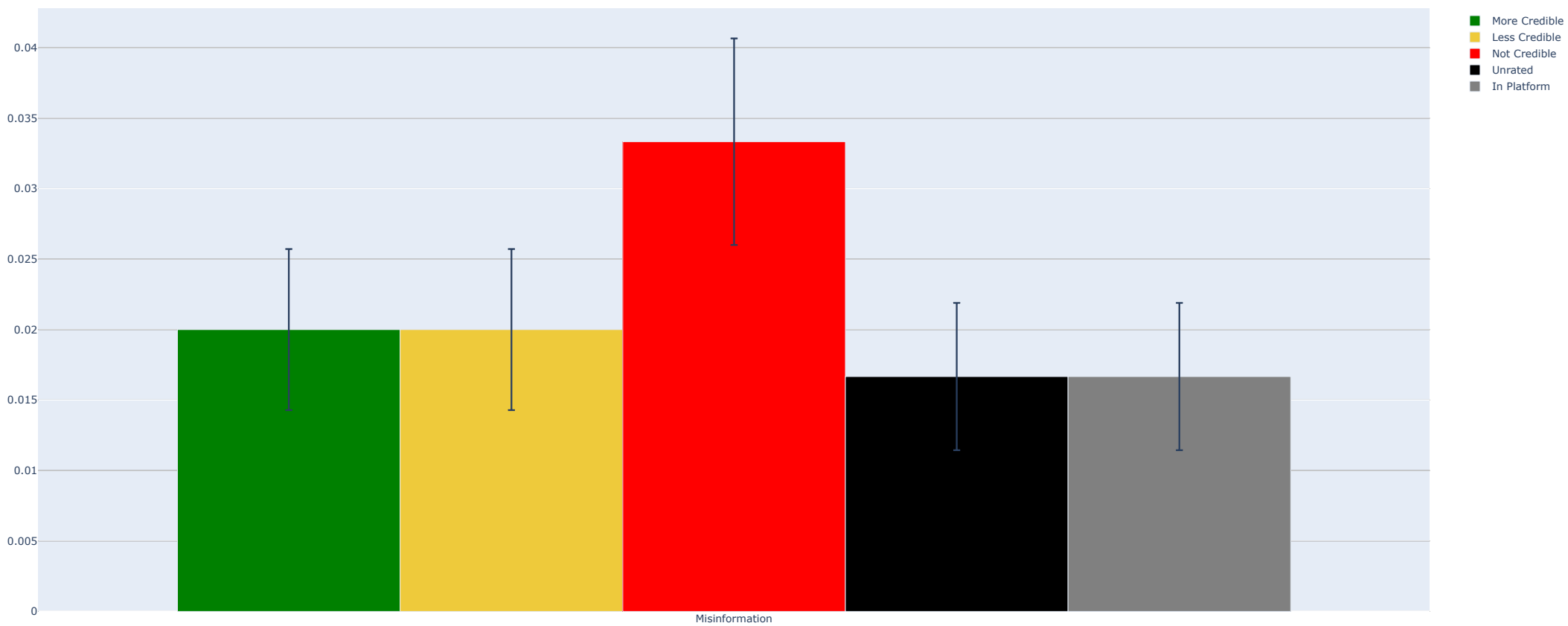

Supplement: S3 Fig — Error bars reflect one standard error. (PDF) [file pone.0261768.s006.pdf]
